# Amperometric Determination of Ascorbic Acid in Pharmaceutical Formulations by a Reduced Graphene Oxide-cobalt Hexacyanoferrate Nanocomposite

**Published:** 2015

**Authors:** Hossein Heli

**Affiliations:** a *Nanomedicine and Nanobiology Research Center, Shiraz University of Medical Sciences, Shiraz, Iran. *; b *Department of Nanomedicine, School of Advanced Medical Sciences and Technologies, Shiraz University of Medical Sciences, Shiraz, Iran.*; c *Department of Medical Physics, School of Medicine, Shiraz University of Medical Sciences, Shiraz, Iran. *

**Keywords:** Reduced graphene oxide, Cobalt hexacyanoferrate, Nanocomposite, Ascorbic acid, Electroanalysis

## Abstract

Investigation of the redox properties of drugs and their determination are performed by electrochemical techniques. Data obtained from electrochemical techniques are often correlated with molecular structure and pharmacological activity of drugs. In this regard, different modified electrodes were applied as sensors for quantification of different drugs.

A nanocomposite of reduced graphene oxide-cobalt hexacyanoferrate was synthesized by a simple precipitation route. Scanning electron microscopy revealed that the nanocomposite comprised nanoparticles of cobalt hexacyanoferrate attached to the reduced graphene oxide nanosheets. A nanocomposite-modified carbon paste electrode was then fabricated. It represented prominent activity toward the electrocatalytic oxidation of ascorbic acid, and the kinetics of the electrooxidation process was evaluated. Finally, an amperometric method was developed for the quantification of ascorbic acid in different pharmaceutical formulations.

## Introduction

Graphene is a single layer of carbon atoms arranged in a hexagonal lattice structure. It is the first two-dimensional crystalline material to be isolated, and owing to its single atom thick nature, it is of immense scientific and applied interest (1-2). Graphene has excellent properties and chemical stability causing great applications in the fields of electronic nanodevices, energy-storage materials, sensors and biosensors (1-6). In the past decades, the electrochemical sensors based on nanostructured materials, such as metal, metal oxide and metal complex nanostructures and carbon nanotubes, have been developed (7-11). Recently, graphene has also been exploited to fabricate sensors and biosensors (3,6,12).

Considerable attention has been attracted to mixed-valence hexacyanometallates with the general formula of M_i_^a+^ [M’(CN)_6_]^b^. n H_2_O due to their attractive properties and applications in different devices (13-15). Among these compounds, cobalt hexacyanoferrate (CoHCF) is of special interest from both fundamental and practical aspects because in this compound both cobalt and iron moieties have two oxidation states of (II) and (III) leading to a multitude of compound stoichiometries and redox states, unique electrochromic properties, reversible thermochromism in wide temperature range, reversible photoinduced magnetization, and good electrocatalytic reactivity towards a variety of biologically important compounds (16).

Ascorbic acid (AA) or vitamin C, as an essential water-soluble vitamin, represents an essential role in many important physiological and metabolic processes such as free radical scavenging, improving immunity and preventing cancer (17). AA which exists widely in food, plant and animal tissues is used in pharmaceutical formulations, cosmetic applications, and as an antioxidant in food (18). AA deficiency leads to the symptoms of scurvy and gingival bleeding; on the other hand, excess AA results in urinary stone, stomach and diarrhea convulsion (19). Determination of AA in the pharmaceutical and food industries is of great importance due to the quality of the products. Up to now, different methods have been proposed for determination of AA in different matrices. In this regard, titration method (20). UV-vis spectrophotometry (21-22). capillary electrophoresis )^23^(, fiber optic reflectance spectroscopy )^24^(, HPLC (25), thermogravimetry (26) and fluorometry (27) have been developed. Recently, there has been a great interest in developing electrochemical sensors for the AA determination. Although AA is electrooxidized on conventional electrode surfaces (28) , this process occurs with high overpotentials resulting in electrode surface fouling by its oxidation intermediate(s)/product(s). Therefore, these electrodes will have poor reproducibility, low selectivity and sensitivity. To overcome these problems, immobilized surface redox species on the electrode surfaces, namely modified electrodes with conducting polymers (29) ,ionic liquid (30) , metal nanoparticles (29), carbon nanotubes (31) , metal complexes (32) and polymeric films (33) have been employed.

 In the present study, the capability of the reduced graphene oxide-CoHCF nanocomposite, prepared by simple precipitation method, as an amperometric sensor for the determination of ascorbic acid in different pharmaceutical formulation, was described. The amperometric method was based on the mediated electro oxidation of ascorbic acid.

## Experimental

*Chemicals and reagents*

 All chemicals used were analytical grade from Merck (Germany) or Sigma (USA) and were used without further purification. Graphite fine powder with an average size of <50 μm was received from Merck (Germany). The AA pharmaceutical forms were obtained from a local drugstore. All solutions were prepared with redistilled water.

*Synthesis of reduced graphene oxide*

Graphene oxide was synthesized from natural graphite using a modified Hummer’s method (34) .In a typical synthesis process, 2.0 g graphite powder was dispersed into 140 mL concentrated sulfuric acid and 1.0 g sodium nitrate was added to the reaction vessel under an ice bath. Then, 6.0 g potassium permanganate was slowly added to the mixture and stirred for 2 h to fully oxidize graphite into graphite oxide. Afterwards, the mixture was diluted with redistilled water. Then, a 5% H_2_O_2_ solution was added to the mixture until the color of the mixture changed into brilliant yellow. The suspension was filtered and the obtained graphene oxide was thoroughly washed by redistilled water. Then the graphene oxide was re-dispersed in redistilled water and exfoliated to generate graphene oxide nanosheets using an ultrasonic bath for 3 hours. The suspension gradually evolved into a brown solution during the ultrasonication, and the bulk graphene oxide powders were transformed into nanosheets. Finally, the exfoliated graphene oxide was reduced to graphene nanosheets by refluxing the graphene oxide solution with hydrazine monohydrate at 100 ºC for 2 hours, and the color of the solution turned into dark black. The final product (the reduced form of graphene oxide) was filtered, washed by redistilled water and ethanol and dried in an oven at 80 ºC.

*Synthesis of reduced graphene oxide-cobalt hexacyanoferrate nanocomposite*

 In order to synthesize the nanocomposite, CoHCF was deposited on the surface of the nanosheets of reduced graphene oxide via a solution-phase deposition. For the deposition, 50 mg graphene was firstly dispersed in 5 mL solution of 2.5 mM Co (NO_3_)_2_ containing 12.4 mM KCl and sonicated for 30 min. Then, 5 mL solution of 12.4 mM K_3_Fe (CN)_6_ was added drop-wise under vigorous stirring. Finally, the mixture was sonicated for 5 min to obtain a precipitate. The precipitate was centrifuged and firstly washed with 3% NaCl solution and then washed three times with redistilled water. The product was dried at room temperature.

*Apparatus*

 Electrochemical measurements were carried out in a conventional three-electrode cell containing 100 mM Na-phosphate buffer solution, pH 7.4 (PBS) powered by an μ-Autolab type III potentiostat/galvanostat (the Netherlands). An Ag/AgCl, 3M KCl, a platinum disk and a modified carbon paste electrode were used as the reference, counter and working electrodes, respectively. The system ran on a PC through GPES 4.9 software. In order to obtain information about the morphology and size of the graphene nanosheets, scanning electron microscopy (SEM) was performed using a X-30 Philips (Germany) instrument. SEM images were analyzed using manual microstructure distance measurement software (Nahamin Pardazan Asia Co., Iran).

*Working electrodes preparation*

Unmodified carbon paste electrode (UCPE) was prepared by hand-mixing carbon powder and mineral oil with an 80/20% (w/w) ratio. The paste was carefully mixed and homogenized in an agate mortar for 20 min. The resulting paste before use was kept at room temperature in a desiccator. The paste was ﬁrmly packed into a cavity (1.0 mm diameter and 2.0 mm depth) at the end of a Teflon tube. Electrical contact was established via a copper wire connected to the paste in the inner hole of the tube. The electrode surface was gently smoothed by rubbing on a piece of weighing paper just prior to use. This procedure was also used to regenerate the surface of the carbon paste electrodes.

 Modified carbon paste electrode (MCPE) was prepared by mixing weighed amounts of carbon powder, Nujol and the nanocomposite with ratios of 60:20:20, %w/w in a similar fashion.

*Analysis of AA pharmaceutical forms*

 Different AA pharmaceutical forms were analyzed including effervescent and chewable tablets, ampoules and sachets. For analysis of the tablets, the average weight of ten tablets from each sample was determined; then, the tablets were finely powdered and homogenized in a mortar. Appropriate and accurately weighed amounts of the homogenized powder were transferred into 100 mL calibrated flasks containing 50 mL of PBS. The contents of the flasks were sonicated for 30 min, and then the undissolved excipients were removed by filtration and diluted to volume with PBS. Appropriate solutions were prepared by taking suitable aliquots of the clear filtrate and diluting them with PBS. For analysis of the sachets, the average weight of ten sachets was determined; then, the sachet contents were finely powdered and homogenized in a mortar and dissolved in 100 mL calibrated flasks containing 50 mL of PBS and diluted to volume with PBS. The ampoule contents were diluted by PBS and directly analyzed.

## Results and Discussion

*Surface morphologies*

**Figure 1:**
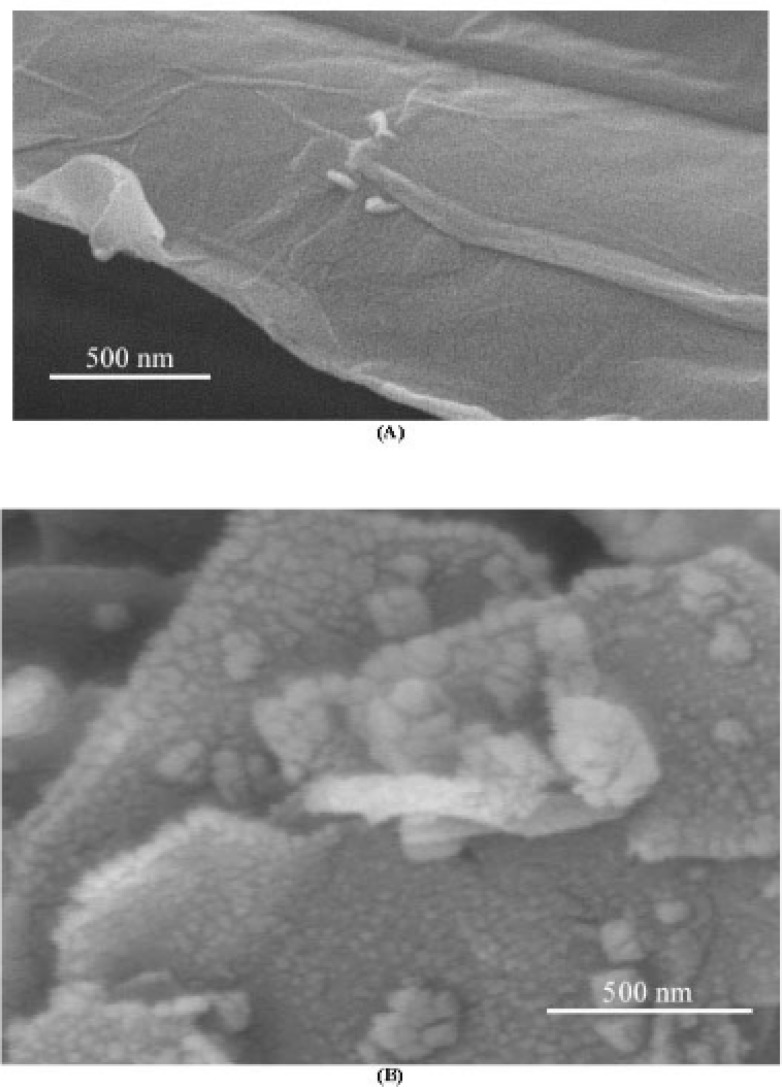
SEM micrographs reduced graphene oxide (A) and the nanocomposite (B).

 The surface morphology of reduced graphene oxide and the nanocomposite was evaluated by SEM micrographs. Figures 1A and 1B represent SEM micrographs of graphene and the nanocomposite, respectively. Graphene comprises a few micrometers dimension nanosheets≈Therefore, the nanocomposite composes the attached CoHCF nanoparticles to the entire surface of graphene.

*Electrocatalytic oxidation of AA on the MCPE surface*

**Figure 2:**
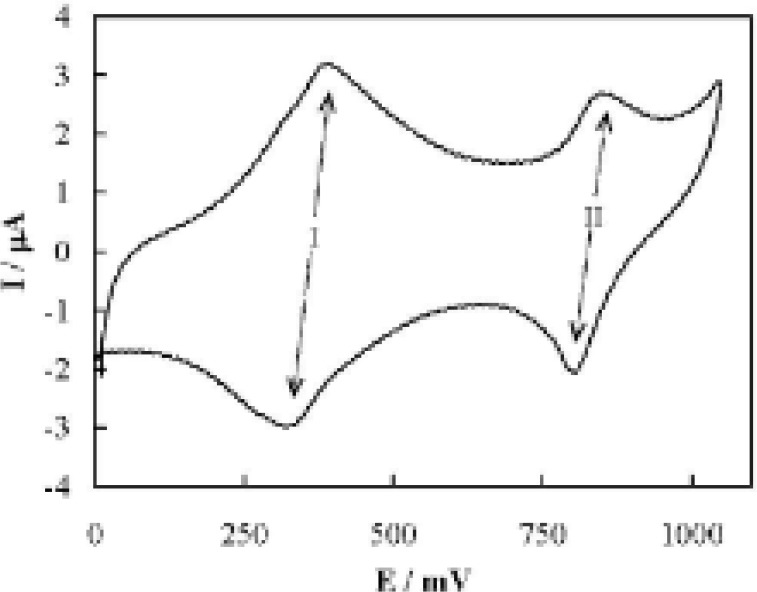
Typical cyclic voltammogram of MCPE recorded in PBS. The potential sweep rate was 50 mV s^-1^.

 Typical cyclic voltammogram of MCPE recorded in PBS at a potential sweep rate of 50 mV s^-1^ is shown in Figure 2. In the voltammogram, two well-defined sets of quasi-reversible redox couples (denoted as couples I and II) appear. The formal potentials of the couples were estimated as 418 and 826 mV, and the ratio of the anodic to the cathodic peak currents for both couples (I_pa_/I_pc_) was almost equal to unity. Based on the direct evidence obtained from combined cyclic voltammetry and in situ IR spectroelectrochemistry techniques (16) X-ray photoelectron and IR spectroscopies (35) UV-vis reflectance spectroelectrochemistry and impedance spectroscopy (36) and the redox processes reported for the low spin iron in Prussian Blue (37) both the cobalt and iron species with the states (II) and (III) are involved in the CoHCF redox reactions. Herein, couples I and II are related to the Co(II)/Co(III) and Fe(II)/Fe(III) transitions, respectively (16,35,36). The redox transitions occurred in couples І and ІІ are:

couple I:

 (3a) $${\mathrm{NaCo}}^{\mathrm{ІІІ}}\left[{\mathrm{Fe}}^{\mathrm{ІІ}}{\left(\mathrm{CN}\right)}_{6}\right]+{\mathrm{Na}}^{+}+e^{-}⇆{\mathrm{Na}}_{2}{\mathrm{Co}}^{\mathrm{ІІ}}{\mathrm{Fe}}^{\mathrm{ІІ}}{\left(\mathrm{CN}\right)}^{6}$$

Or

 (3b) $${\mathrm{Na}}_{0.4}{\mathrm{Co}}_{1.3}^{\mathrm{III}}\left[{\mathrm{Fe}}^{\mathrm{ІІ}}{\left(\mathrm{CN}\right)}_{6}\right]+{\mathrm{Na}}^{+}+e^{-}⇆{\mathrm{Na}}_{1.4}{\mathrm{Co}}_{1.3}^{\mathrm{II}}{\left(\mathrm{CN}\right)}^{6}$$

couple II:

 (4a) $${\mathrm{Co}}_{1.5}^{\mathrm{II}}\left[{\mathrm{Fe}}^{\mathrm{ІIІ}}{\left(\mathrm{CN}\right)}_{6}\right]+{\mathrm{Na}}^{+}+e^{-}⇆{\mathrm{NaCo}}_{1.5}^{\mathrm{II}}[{\mathrm{Fe}}^{\mathrm{II}}{\left(\mathrm{CN}\right)}_{6}]$$

or

 (4b) $${\mathrm{Co}}^{\mathrm{III}}\left[{\mathrm{Fe}}^{\mathrm{ІIІ}}{\left(\mathrm{CN}\right)}_{6}\right]+{\mathrm{Na}}^{+}+e^{-}⇆{\mathrm{NaCo}}^{\mathrm{III}}[{\mathrm{Fe}}^{\mathrm{II}}{\left(\mathrm{CN}\right)}_{6}]$$

It should be noted that the redox reactions of metal hexacyanoferrates (MHCFs) involve cation insertion into the MHCF solid state and electron transfer within MHCF (38).

**Figure 3:**
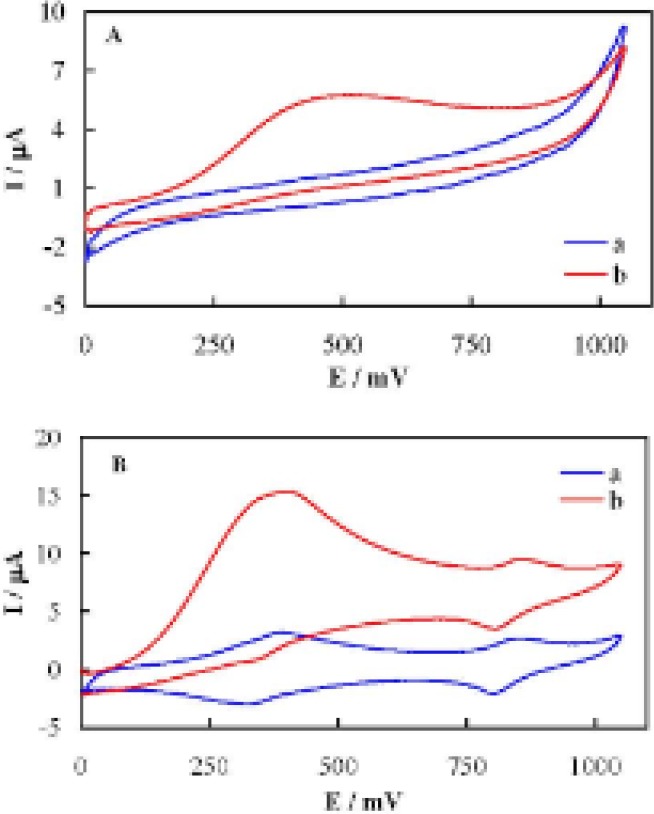
Cyclic voltammograms of UCPE (A) and MCPE (B) recorded in the absence (curve a) and presence (curve b) of 3.75 10^-^^4^ M AA in PBS.

 Cyclic voltammograms of UCPE and MCPE recorded in the absence and presence of 3.7510^-^^4^ M AA in PBS is shown in Figure 3. AA was oxidized on the UCPE surface and generated a broad anodic peak. In the case of MCPE, AA caused an increment in the anodic current of couple I and a decrement in the corresponding cathodic current occurred while the peak currents of couple II almost remained constant (concerning the currents correcting with respect to the baseline current). The anodic charge of couple I in the presence of AA is >10 times higher than the corresponding cathodic charge. The anodic and cathodic charges in the absence of AA are almost the same. These results indicate that AA was oxidized by the Co(III) moiety during couple I through a cyclic mediation redox process (an electrocatalytic oxidation mechanism). Different valence cobalt species are immobilized on the electrode surface, and the species with the higher valence (Co(III)) oxidizes AA via a chemical reaction followed by generation of low-valence (Co(II)) species. Then, the high-valence species is regenerated through the external electrical circuit. Accordingly, AA is oxidized via an electrocatalytic (EC’) process. Moreover, the current in the reverse sweep indicates that the reaction of AA with the high-valence species is the rate-determining step of the oxidation process. It should be noted that although AA is electro reactive on the UCPE surface via a direct electron transfer process, it is oxidized at lower potentials and generates higher electro oxidation current on the MCPE surface. Therefore, MCPE better oxidizes AA than UCPE from both thermodynamic and kinetic points of view. Based on the presented results, the electrocatalytic oxidation of AA on the MCPE surface can be expressed as:

$\mathrm{Co}\left(\mathrm{III}\right)+e⇆\mathrm{Co}(\mathrm{II})$

 (12)

(13) CoIII+AA Kcat→Dehydroascorbic acid + Co(ΙI)

*Kinetics of the AA electrooxidation process*

**Figure 4:**
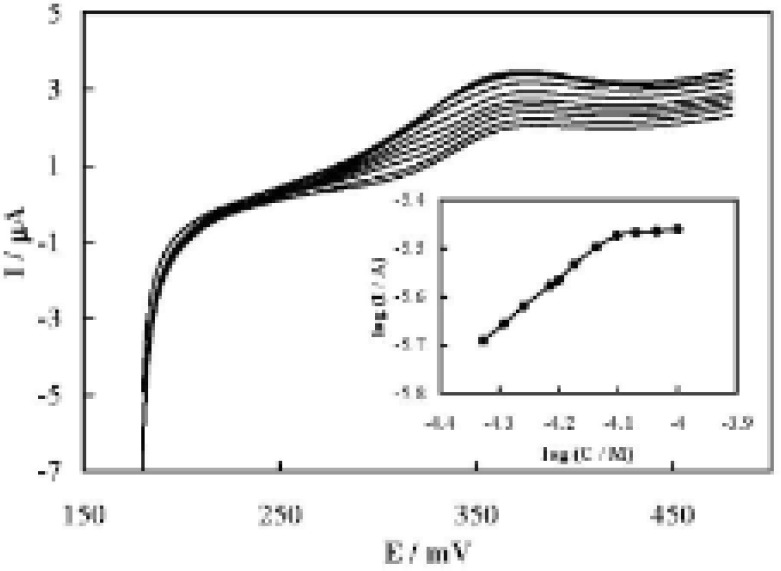
Linear sweep voltammograms of different concentrations of AA of 47, 51, 55, 61, 63, 67, 73, 79, 85, 92.1 and 100 μM, using MCPE in the potential range of couple I. The potential sweep rate was 35 mV s^-1^. Inset: The dependency of the logarithm of the peak current on the logarithm of AA concentration.

Figure 4 shows linear sweep voltammograms recorded at different concentrations of AA, and the dependency of logarithm of anodic peak current for couple I on the logarithm of AA concentration. The concentration dependency of the anodic peak current on the bulk concentration of AA was linear up to approximately 79 μM and then it reached a plateau. This dependency indicates surface saturation kinetics and is close to the domination of a Michaelis-Menten reaction mechanism (39) .In addition, the reaction order can be estimated based on the slope of the line in the inset to be the first order. It is close to the Michaelis-Menten prediction (39-40).

**Figure 5:**
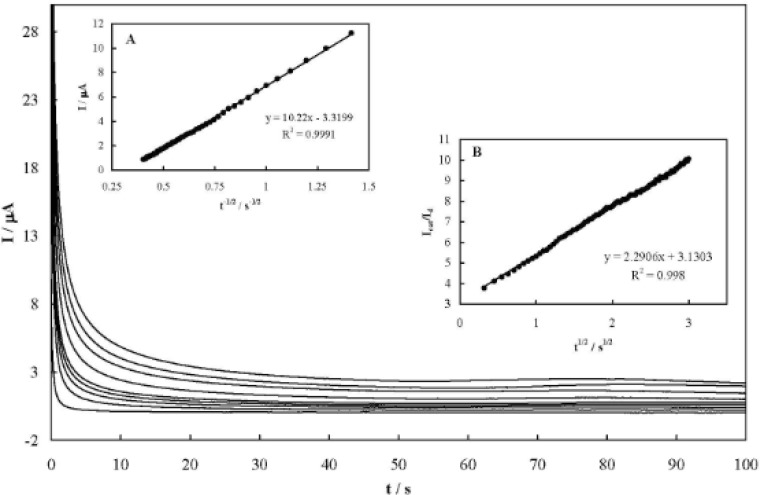
Chronoamperograms recorded in the absence and presence of AA over the concentration range of 0, 0.5, 1.0, 2.0, 5.0, 13.0, 20.0, 25.0, 30.0 mM. The potential step was 430 mV, corresponding to the anodic couple I. Inset A: Dependency of the net transient current on the minus square roots of time. Inset B: dependency of I_cat_/I_d_ on square roots of time.

 Chronoamperometry technique was also employed to study the mass transfer kinetics and obtain the heterogeneous catalytic rate constant in the electro catalytic mechanism )41(. Chronoamperograms of MCPE in the absence and presence of different AA concentrations are represented in Figure 5. The net transient current varied linearly with the minus square roots of time (Figure 5, inset A). This confirms that the electrooxidation process is diffusion-controlled in the bulk of solution. Using the slope of this line, the diffusion coefficient of AA can be obtained based on Cottrell’s equation (41) : 

 (25) $$I={\mathrm{nFAD}}^{1/2}C^{-1/2}t^{-1/2}$$

Where I is the net transient current, D is the AA diffusion coefficient, and C is the AA concentration. The mean value of the AA diffusion coefficient was found to be 1.42(±0.06)10^-6^ cm^2^ s^-1^. Chronoamperometry can also be used to evaluate the catalytic rate constant using the following equation:

(26) $$\frac{I_{\mathrm{cat}}}{I_{d}}=\zeta^{1/2}[\pi^{\frac{1}{2}}\mathrm{erf}\left(\zeta^{\frac{1}{2}}\right)+\exp/\zeta^{1/2}]$$

Where I_cat_ and I_d_ are the currents in the presence and absence of AA, respectively, ζ=k_cat_Ct is the argument of the error function, k_cat_ is the catalytic rate constant, and t is the elapsed time (41) .In the condition ζ>1.5, erf(ζ^1/2^) becomes almost unity and the above equation is simplified as:

The plot of I_cat_/I_d_ versus t^1/2^ is shown in Figure 5, inset B, and from its slope, the mean value of the catalytic rate constant was obtained as k_cat_=8.87(±0.21)10^4^ cm^3^ mol^-1^ s^-1^.

*Amperometric determination of AA*

 In order to develop an electrochemical sensor for the analysis of AA, an amperometric method was developed. Typical amperometric signals obtained during successive addition of AA to PBS are shown in Figure 6. Gentle stirring for a few seconds was needed to promote solution homogenization after each injection. The electrode response was quite rapid and proportional to the AA concentration. The corresponding calibration curve for the determination of AA is shown in Figure 6, inset. The limits of detection (LOD) and quantification (LOQ) of the procedure were obtained as 3SD/m and 10SD/m, respectively, where SD is the standard deviation of the intercept and m is the slope of the calibration curve )^42^(. The determined parameters for the calibration curves of AA are reported in Table 1.

**Figure:**
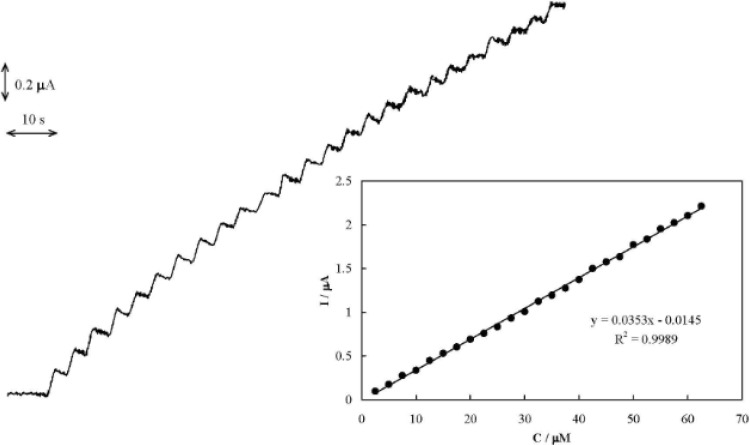
Typical amperometric signals obtained during successive increment in the AA concentration. Inset: The calibration curve.

**Table 1:** The determined parameters for the calibration curves of AA and accuracy and precision (n=3) using MCPE.

Linear range (µM)	2.5-62.5
Slope (mA M^-1^)	35.3±0.3
Intercept (μA)	-0.015±0.006
R^2^	0.999
LOD (µM)	0.29
LOQ (µM)	0.97
RSD (%)	3.48
Bias (%)	3.18

*Selectivity, durability and stability*

 Selectivity of the amperometric sensor for the assay of AA was examined in the presence of some common excipients/ingredients in the same ratios usually used in pharmaceutical preparations. These interferences/ingredients include microcrystalline cellulose, magnesium stearate, phenyl alanine, aspartame, tartaric acid, sodium bicarbonate, citric acid, calcium carbonate, sucrose crystalline, polyethylene glycol, acetylsalicylic acid, acetylcysteine, sorbitol, lactose, and sodium saccharin. The results showed no significant interference from these compounds, because they are not electroreactive at the working potential, pH and the modified electrode. Therefore, the procedure is able to assay the drug in the presence of excipients/ingredients and, hence, it can be considered selective.

 In order to verify the durability and long-term stability of MCPE, 50 consecutive cyclic voltammograms using this electrode were recorded in PBS. The results showed that the peak currents changed slightly (<5%). In addition, the electrode was stored in PBS when not in use, retaining its electrochemical reactivity for four weeks.

*Determination of AA in pharmaceutical forms*

 The applicability of the proposed amperometric method for the sample dosage form was examined by analyzing different AA pharmaceutical forms. It was found that the drug concentrations determined using this method were in good agreement with the reported values, as reported in Table 2. In order to evaluate the accuracy of this method and to know whether the excipients in pharmaceutical dosage forms show any interference with the analysis, the proposed amperometric method was checked by recovery experiments using the standard addition method. After addition of known amounts of pure AA to various pre-analyzed formulations of AA, the mixtures were analyzed by the proposed method. The recovery values of AA were calculated using the corresponding regression equations of previously plotted calibration plots. The results of recovery experiments using the developed assay procedure are also presented in Table 2. The results indicate the lack of interference from commonly encountered pharmaceutical excipients used in the selected formulations. Therefore, the method can be applied to the determination of AA in pharmaceutical forms without any interference from inactive ingredients. A comparison between some electro analytical methods of determination of AA is presented in Table 3. This indicates that the nanocomposite can determine AA with a low detection limit in pharmaceutical forms.

**Table 2:** Determination and recovery of AA in pharmaceutical forms.

**Sample Type**	**Amount labeled**	**Amount added**	**Amount found**	**Recovery (%)**	**RSD (%)**	**Bias (%)**
Effervescent tablet-1	1000 mg	-	986.1 mg	98.61	3.79	-1.39
Effervescent tablet-1	-	1000 mg	990.3 mg	99.03	2.95	-0.97
Effervescent tablet-1	-	1000 mg	988.7 mg	98.87	2.61	-1.13
Effervescent tablet-2	500 mg	-	485.2 mg	97.04	2.97	-2.96
Effervescent tablet-2	-	500 mg	489.1 mg	97.82	4.88	-2.18
Effervescent tablet-2	-	500 mg	498.9 mg	99.78	3.50	-0.22
Effervescent tablet-3	240 mg	-	243.1 mg	101.29	3.97	1.29
Chewable tablet-1	500 mg	-	483.5 mg	96.7	3.10	-3.30
Chewable tablet-1	-	500 mg	493.4 mg	98.68	3.17	-1.32
Chewable tablet-1	-	500 mg	499.1 mg	99.82	4.16	-0.18
Chewable tablet-2	250 mg	-	243.8	97.52	2.85	-2.48
Ampoule	500 mg/5 mL	-	498.7	99.74	4.06	-0.26
Sachet	75 mg	-	74.5	99.33	3.52	-0.67

**Table 3:** Comparison of some electroanalytical methods of determination of AA.

**Type of electrode**	**Pharmaceutical sample**	**Linear range / ** **μ** **M**	**LOD / ** **μ** **M**	**Reference**
CoHCF	Tablet	55.2-32300	33.3	(43)
PtAu film	Injection solution, tablet	103-1650	103	(44)
Carbon-nanofiber	-	2.0-64	2.0	(45)
Poly (vinyl alcohol)	-	10.0-250	7.6	(46)
Tin hexacyanoferrate	-	400-50000	-	(47)
Mesoporous carbon/Nafion		40-800	0.5	(48)
Oxidized glassy carbon electrode	-	197-988	-	(49)
Silver hexacyanoferrate nanoparticles	-	4.0-78	0.42	(50)
Nickel hexacyanoferrate-modified Al	Tablet, ampoule, syrup	2-200	2	(51)
Multi-walled carbon nanotubes-chitosan	Tablet	1-400	0.8	(52)
Co(II) phthalocyanine-multi-walled carbon nanotubes	-	10-2600	1.0	(53)
Single-walled carbon nanotube-modified carbon-ceramic	Tablet	5.0-700.0	3.0	(54)
Pyrolytic graphite electrodes modified in dopamine solution	-	25-500	13	(55)
Graphene oxide-CoHCF nanocomposite	Tablet, ampoule, sachet	2.5-62.5	0.29	(This work)

## Conclusion

 A nanocomposite of reduced graphene oxide-cobalt hexacyanoferrate was synthesized via a precipitation route. Cobalt hexacyanoferrate was deposited as nanoparticles of 50 nm on the graphene surface. This method would be useful for the synthesis of other MHCFs-graphene nanocomposites. The electrochemical behavior of the nanocomposite was studied by cyclic voltammetry and the kinetics of charge transfer and mass transport processes within the nanocomposite were evaluated. The nanocomposite has been applied to fabricate a modified carbon paste electrode and employed as an efficient electrocatalytic transducer for the electrocatalytic oxidation of AA. The AA electrooxidation process was diffusion-controlled, and the catalytic rate constant and diffusion coefficient involved in the reaction were reported. The amperometric responses of the electrode indicated that AA can be determined by high sensitivity in pharmaceutical formulations. The electrode can be applied in routine analysis of AA and as a detector in detecting systems.
